# Systems Biology Approach to Bioremediation of Nitroaromatics: Constraint-Based Analysis of 2,4,6-Trinitrotoluene Biotransformation by *Escherichia coli*

**DOI:** 10.3390/molecules22081242

**Published:** 2017-08-14

**Authors:** Maryam Iman, Tabassom Sobati, Yunes Panahi, Meysam Mobasheri

**Affiliations:** 1 Chemical Injuries Research Center, Baqiyatallah University of Medical Sciences, 1477893855 Tehran, Iran; m-iman@alumni.tums.ac.ir (M.I.); yunespanahi@yahoo.com (Y.P.); 2 Department of Pharmaceutics, School of Pharmacy, Baqiyatallah University of Medical Sciences, 1477893855 Tehran, Iran; 3 Young Researchers and Elite Club, Islamic Azad University, 46115655 Tehran, Iran; tmsobati@gmail.com; 4 Department of Biotechnology, Faculty of Advanced Sciences & Technology, Pharmaceutical Sciences Branch, Islamic Azad University (IAUPS), 194193311 Tehran, Iran

**Keywords:** nitroaromatics, 2,4,6-trinitrotoluene, environment, bioremediation, biotransformation, bio-degradation, constraint-based analysis, systems biology, metabolic engineering

## Abstract

Microbial remediation of nitroaromatic compounds (NACs) is a promising environmentally friendly and cost-effective approach to the removal of these life-threating agents. *Escherichia coli* (*E. coli*) has shown remarkable capability for the biotransformation of 2,4,6-trinitro-toluene (TNT). Efforts to develop *E. coli* as an efficient TNT degrading biocatalyst will benefit from holistic flux-level description of interactions between multiple TNT transforming pathways operating in the strain. To gain such an insight, we extended the genome-scale constraint-based model of *E. coli* to account for a curated version of major TNT transformation pathways known or evidently hypothesized to be active in *E. coli* in present of TNT. Using constraint-based analysis (CBA) methods, we then performed several series of in silico experiments to elucidate the contribution of these pathways individually or in combination to the *E. coli* TNT transformation capacity. Results of our analyses were validated by replicating several experimentally observed TNT degradation phenotypes in *E. coli* cultures. We further used the extended model to explore the influence of process parameters, including aeration regime, TNT concentration, cell density, and carbon source on TNT degradation efficiency. We also conducted an in silico metabolic engineering study to design a series of *E. coli* mutants capable of degrading TNT at higher yield compared with the wild-type strain. Our study, therefore, extends the application of CBA to bioremediation of nitroaromatics and demonstrates the usefulness of this approach to inform bioremediation research.

## 1. Introduction

Nitroaromatic compounds (NACs) are major environmental contaminants with high toxicity to living systems and human health [1,2,3]. Intensive industrial application of NACs during the past decades has resulted in their widespread release in the environment [1]. Because of their established toxic, mutagenic, and carcinogenic potential [1,4,5,6] NACs are declared as priority pollutants by regulatory bodies such as the United States Environmental Protection Agency [1,7]. Due to its superior physicochemical properties, 2,4,6-trinitrotoluene (TNT) is the most widely used NAC in the industry and hence has the largest contribution to environmental contamination [8,9]. The requirement for clean-up of TNT-polluted regions has motivated development and examination of several remediation processes including composting [10], incineration [11], and chemical oxidation [12]. These methods, however, suffer from one or more drawbacks such as high cost, incomplete degradation, generation of potentially more toxic by-products, and health and safety risks to workers [2,6,13]. These limitations call for renewed efforts for development of more efficient approaches.

Recent evolution of microorganisms in the TNT-contaminated soils has led to the emergence of microbes with the ability to not only survive in presence of TNT but also to degrade it and/or metabolize it as a nutrient source. Along with continued discovery and characterization of such “specialist” microorganisms, the microbial remediation shows increasing promise as an environmentally sound and economic viable TNT degradation approach [2,6,8,13].

The advantages realizable by microbial degradation of TNT have prompted extensive research on different aspects of the issue, including capabilities of degrader microorganisms [14], enzymes [15,16,17,18,19] and catabolic pathways [2,9,20] involved in co-metabolic transformation and/or mineralization of TNT, and effect of culture conditions [21,22,23,24,25] on bioremediation performance. Particularly, a large body of research is devoted to isolation and characterization of TNT-degrading microbes [3,6,9,13,26]. *E. coli* is one of the microorganisms that has shown remarkable TNT biotransformation capability [27,28,29,30]. *E. coli* possesses multiple enzymes to attack TNT, including nitroreductases capable of reducing the TNT nitro groups and N-ethylmaleimide (NEM) involved in TNT denitration [28]. *E. coli* is able to derive assimilatory ammonium from TNT, placing it among the limited number of microbes capable of growing on TNT as the sole nitrogen source [27,28]. In addition, the organism has shown significant tolerance against TNT toxicity while maintaining the biotransformation capability at relatively high TNT concentrations [31,32]. These characteristic advantages together with the wealth of information on physiology and molecular biology of *E. coli*, highlight this organism as a promising biocatalyst for TNT bioremediation processes.

An efficient bioremediation process requires the degrader microbe(s) to be improved for high yield and productivity. In the post-genomic era, development of microbial biocatalysts relies heavily on genome-wide knowledge of cellular metabolism obtainable from of holistic analysis of omic data [33,34,35]. Systems-level understanding of flexibility and bioenergetic control of metabolic flux distribution enables efficient engineering of both strain and process for the biotechnological goal of interest [34,36]. One of the successful approaches to systems study of microbial metabolism is constraint-based analysis (CBA) [37]. Built upon principles of linear programming, CBA enables quantitative analysis of cellular metabolism by constraining the phenotype space to evident physico-biological constraints and optimal metabolic operation [38]. CBA of genome-scale metabolic models (GSMMs) has been successfully applied to study bioremediation capability of several species, including *Pseudomonas putida* KT2440 (in degradation of aromatics) [39], *Dehalococcoides* (in detoxifying chloro-organic pollutants) [40], *Geobacter metallireducens* (in reduction of toxic metals) [41], and *Rhodococcus erythropolis* (in desulfurization) [42].

While the need for high-performance NACs biodegradation process persists [6,9], the potential of CBA in catalysing the progress in the field remains untapped. Therefore, in the present study we used CBA to profile the metabolic capacity of *E. coli* in biotransformation of TNT. In doing so, we extended the most comprehensive high quality GSMM of *E. coli*, iJO1336 [43], to account for the known or evidently hypothesized pathways involving in TNT degradation by this organism [1,2,3,9,13,27,28,29,30,44,45]. The extended model was then analysed by a set of CBA methods [37], including flux balance analysis (FBA), flux variability analysis (FVA), Monte Carlo flux sampling [46], robustness analysis (RA), and phenotype phase plane (PhPP) analysis for network-level characterization of TNT transformation in *E. coli*.

We report our results accompanied by the relevant discussion as the following: firstly a detailed analysis of the efficiency of each individual TNT degradation pathway will be presented. Then, the capacity of *E. coli* when all pathways are present and the flexibility of flux distribution under different growth conditions will be evaluated. Subsequently the extended model will be used to predict TNT depletion profile in the *E. coli* batch growth culture. Next we analyse the influence of bioprocess conditions, including aeration regime, TNT concentration, cell density, and carbon source on biotransformation yield/productivity. Ultimately, we introduce a series of in silico designed *E. coli* mutants potentially more efficient in TNT degradation compared with the wild-type strain. The implication of our study for future research in microbial remediation of NACs will also be discussed.

## 2. Results and Discussion

### 2.1. Characterization of E. coli TNT Biotransformation Capacity

#### 2.1.1. Nitroreduction Pathway

##### FBA and FVA

Reductive transformation to nitroso and hydroxylaminotoluene is the primary step in most known TNT biodegradation pathways [8,9]. Initial metabolites successively derived from TNT include nitrosodinitrotoluenes (NDTs), hydroxylaminodinitrotoluenes (HADNTs) and amino-dinitrotoluenes (ADNTs) [8,9]. The latter group of compounds may be transformed to more reduced metabolites including diaminomononitrotoluenes (DANTs) and ultimately triaminotolene (TAT) [1,2,8,9,13]. Several bacterial species with the ability to reduce TNT nitro groups have been described [1,3,26]. *E. coli* has proven ability to transform TNT to ADNTs and DANTs under both aerobic and anaerobic growth conditions [13,30,32,47,48]. TAT has also been detected in cell extract of *E. coli* grown anaerobically under a hydrogen atmosphere [49].

**Figure 1:**
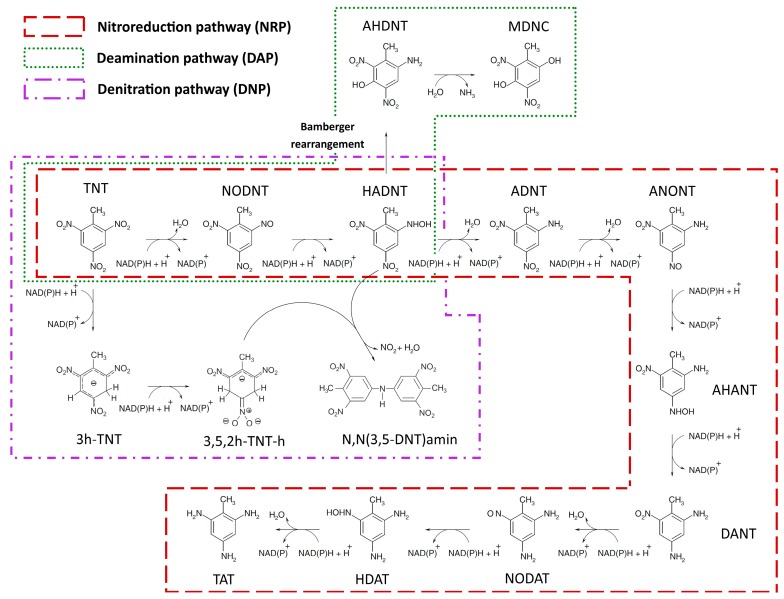
The representative TNT biotransformation pathways known or evidently hypothesized to operate in *E. coli* in the presence of TNT [1,2,3,9,13,27,28,29,30,44,45].

**Figure 2:**
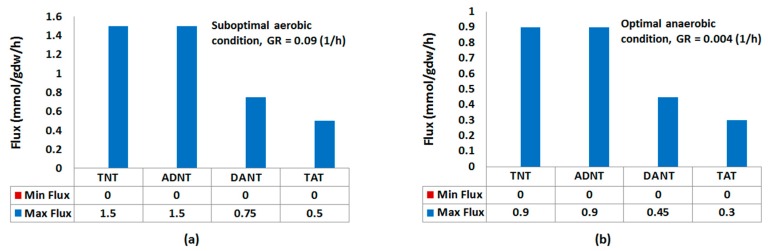
Minimum and maximum feasible TNT uptake and reduced metabolite formation rates through activity of nitroreduction pathway under (**a**) aerobic and (**b**) anaerobic conditions for a glucose uptake rate of 250 mg/gdw/h.

To explore this capability of *E. coli* in the light of CBA, we incorporated reactions of nitroreduction pathway (NRP) up to TAT formation (Figure 1) into the genome-scale metabolic model (GSMM) of the strain, iJO1366. We then simulated the growth of *E. coli* on glucose and ammonium as the carbon and nitrogen sources, respectively, in presence of TNT at glucose uptake rate (GUR) of 250 mg/gdw/h. Optimal aerobic growth as simulated by flux balance analysis (FBA) did not allow flux through TNT biotransformation. The result of FBA was confirmed by flux variability analysis (FVA). This result suggests that experimentally observed nitroreduction of TNT would have occurred under suboptimal growth conditions [27]. To apply the suboptimal conditions in silico, we constrained the growth rate to an upper limit of 70% of the maximum. We then simulated the growth under suboptimal aerobic and optimal anaerobic conditions. Figure 2 shows the minimum and maximum TNT uptake rate (TNTUR) and fluxes through 2-amino-4,6-dinitrotoluene (ADNT), 2,4-diamino-6-nitrotoluene (DANT), and 2,4,6-triaminotoluene (TAT) under both conditions. Although the minimum rates of TNT uptake and reduced metabolite formation at both aeration conditions are no more than zero, the positive maximum of the corresponding rates reflect the capability of *E. coli* in transforming TNT to reduced derivatives aerobically and anaerobically [27,28,29,30,32]. The maximum TNTUR and maximum fluxes through the nitroreduction products are higher at anaerobic condition. This finding together with infeasibility of nitroreduction activity under optimal aerobic regime replicate the broadly observed higher efficiency of TNT nitroreduction under anoxic condition [1,9,13,30].

As seen from Figure 2, under both aeration regimes the maximum flux through the nitroreduction products decreases proportionally to the amount of NADPH required for their formation. This in accord with experimental findings [30] which imply that the degree of TNT reduction is controlled by the availability and distribution of reducing equivalents.

##### Sampling of Flux Space

Although a reaction flux can take any arbitrary value across FVA-determined range without affecting growth, the probability distribution of flux within the allowable interval is not uniform. In other words, the flux of a particular reaction may not take all feasible values with similar probability. The actual arrangement of flux through metabolic network reactions is highly influenced by the topology of the flux space. Uniform random sampling of the flux space thus can be used to obtain the probability distribution of a flux of interest within the feasible range, and thereby the most likely value of the flux [46] (see Materials and Methods).

Table 1 present the average values of TNTUR and nitroreduction fluxes obtained from flux sampling under aerobic (suboptimal) and anaerobic regimes. As seen, during the aerobic growth, mean and median of fluxes through the nitroreduction pathway are very small tending to the lower limit (zero). However, under anaerobic condition, the mean TNTUR and ADNT formation flux approach the midpoint of the allowable range. Also average flux through DANT, though small relative to ADNT, is an order of magnitude larger than that under aerobic conditions. Sampling of the solution space at various thresholds of suboptimality produced similar patterns. These data reveal that the higher efficiency of nitroreduction under anaerobic regime is not only due to lower redox potential, but is also fundamentally supported by the structure of flux space. In addition, the very small average flux of TAT formation irrespective of the aeration mode is correlated with lack of detectable amounts of TAT in TNT amended *E. coli* cultures [27,29,30].

##### Robustness Analysis

To further characterize the flux-level properties of NRP, a robustness analysis of the pathway was conducted. Robustness analysis (RA), is a CBA technique for estimating the amount of change in the optimal value of an objective function per unit of change in a relevant variable [50] (see Experimental Section). We used RA to identify how optimal growth will be influenced by every unit of increase in formation rates of ADNT, DANT, and TAT and in turn how these rates are affected by every unit of increase in TNTUR.

**Figure 3:**
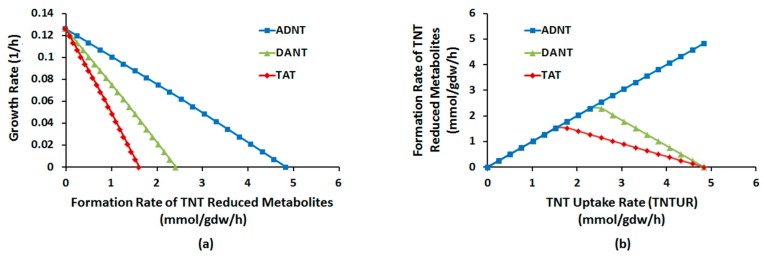
Robustness diagram: (**a**) optimal growth rate (GR) as function of fluxed through TNT (nitro)reduced metabolites; (**b**) optimal flux through TNT (nitro)reduced metabolites as a function of TNT uptake rate (TNTUR).

Figure 3 present the results of RA under aerobic conditions. As seen from Figure 3a, optimal growth has a negative correlation with flux through TNT-derived compounds. It is observed that the amount of reduction in growth rate for every unit of flux through ADNT is approximately half and one third as high as that through DANT and TAT, respectively. Therefore, correlated with findings from FBA and FVA, RA shows that sensitivity of growth to production of each reduced metabolite is proportional to NADPH demand for its formation.

Figure 3b shows robustness of formation of reduced metabolites against TNTUR. As seen, for DANT and TAT there is threshold of TNTUR at which the increasing trend of flux stops and then begins to decrease (2.3 and 1.6 mmol/gdw/h, respectively). For ADNT, however, no such threshold exists and the flux through this compound monotonically increases with TNTUR up to the ultimate cell capacity. Therefore, unlike ADNT, formation rates of DANT and TAT show inherent limitations to augment with TNTUR. Qualitatively similar results were also observed under anaerobic conditions. These observations along with results FBA, FVA, and flux sampling provides a systems-level explanation for incomplete nitroreduction of TNT in most bacterial cultures [9,13,26,27,30,44,51,52].

#### 2.1.2. Deamination Pathway

Apart from TNT nitroreduction, *E. coli* has the ability to grow on TNT as a sole nitrogen source [27,28]. Based on the best available evidence, the assimilatory utilization of TNT by *E. coli* is initiated by transformation to HADNTs through the activity of nitroredutases, which in turn suggestively undergoes a Bamberger-rearrangement followed by deamination by a putative mutase and ammonium lyase, respectively, ending up with assimilatory ammonium release [27,28,53,54]. To examine the efficiency of assimilatory TNT degradation by *E. coli* we constructed a representative elementary and charge balanced version of the deamination pathway (DAP) (Figure 1) in the metabolic model, and performed the simulation of growth in a NH4-free TNT-supplemented glucose-limited culture, at GUR = 250 mg/gdw/h. Under aerobic regime by uptaking 1.15 mmol/gdw/h of TNT and assimilation of 33% of its nitrogen, a GR of ~0.109/h was observed which is 85% of the corresponding value in NH_4_-supplied culture (GR = 0.13/h). The lower GR during growth on TNT can be explained by the two reducing equivalents required for release of each NH_4_ molecule via DAP vs. free access to NH_4_ in ammonium-supplemented culture. Under suboptimal condition, at GR of 0.076/h, TNTUR was found to vary within the range of 0.8–3.36 mmol/gdw/h averaging at 0.93 ± 0.15 mmol/gdw/h (median: 0.89 mmol/gdw/h). Given that the GR is close to the corresponding value during NRP activity, the significantly higher mean TNTUR shows the greater efficiency of DAP relative to NRP.

Although not explored experimentally, we also evaluated the potential capability of *E. coli* to grow anaerobically on TNT. As predicted by FVA, at TNTUR ranging within 1.30–5.23 mmol/gdw/h an optimal GR of 0.011/h could be achieved, being ~10 fold lower than in the aerobic culture. While the maximum feasible TNTUR is 4.5 fold higher compared with aerobic cultivation, flux sampling identified an average TNTUR of 2.68 ± 0.22 mmol/gdw/h (median: 2.67 mmol/gdw/h), corresponding to a nitrogen assimilation of 1.4%. Based on these data, we concluded a generally lower efficiency of DAP under anoxic vs. oxic condition. Nonetheless, when comparison is made with non-assimilatory anaerobic activity of NRP (FBA and FVA results), DAP shows considerably higher efficiency.

#### 2.1.3. Denitration Pathway

Two previous studies provided evidence that assimilatory utilization of TNT by *E. coli* is accompanied by release of TNT-derived nitrite [27,28]. The only proven pathway for denitration of TNT is abiotic condensation of HADNTs and the protonated Meisenheimer dihydrides resulting in the formation of secondary diarylamines accompanied by nitrite release [9]. Although *E. coli*’s assimilatory nitrite reductase is non-functional under an aerobic regime [55] and thus the TNT-derived nitrite accumulates, there is evidence that denitration activity contributes to aerobic TNT degradation [28]. To study the efficiency of TNT degradation through nitrite release, we incorporated a curated representative denitration pathway (DNP) into the *E. coli* model, followed by simulation of growth in a glucose-limited medium containing both NH4 and TNT (GUR = 250 mg/gdw/h). While under optimal aerobic condition (GR = 0.13/h), neither TNT uptake and nor DNP activity was observed, suboptimal condition (70%; 0.088/h), allowed a maximum TNTUR of 2.26 mmol/gdw/h with concomitant release of nitrite up to 3.94 mmol/gwd/h. Sampling of flux space determined the average TNTUR to be 0.025 ± 0.088 mmol/gwd/h (median 0.07 mmol/gdw/h), predicting an order of magnitude lower activity of DNP than DAP under aerobic regime. This activity however is 2.5 fold as high as that of NRP at corresponding cultivation condition.

Unlike aerobic conditions, under an anoxic regime *E. coli* possesses an active assimilatory nitrite reductase [55] allowing use of NO_2_ as a nitrogen source. The potential contribution of this route to TNT degradation capacity of *E. coli* has not been explored experimentally, thus in silico analysis can yield useful insights. According to our simulation, by uptaking 3.84 mmol/gdw/h of TNT, cell is able to grow as high as 0.038 /h with concomitant ammonium exertion of 1.52 mmol/gdw/h. The observed GR is 10-fold that during anaerobic growth on ammonium and 3.45-fold of that during anaerobic TNT assimilation through DAP. TNTUR through anaerobic DNP activity is 1.4-fold higher than the corresponding average value during anaerobic DAP activity. Also a nitrogen balance revealed that 3.4% of TNT nitrogen is assimilated through DNP which is 2.4-fold of that possible through anaerobic DAP. These data consistently indicate that DNP has the highest anaerobic TNT biotransformation efficiency among other alternative routes, suggesting the potentially significant contribution of this pathway in *E. coli* nitroaromatic degradation capability.

#### 2.1.4. Interactions between Multiple TNT Biotransformation Pathways

Above we analysed the efficiency of three major TNT biotransformation pathways of *E. coli* individually. In practice, however, these pathways may function simultaneously and interact during metabolism of TNT as implicated from presence of TNT-derived NO_2_, NH_4_ and nitroaminotoluene products in *E. coli* cultures [27,28]. Therefore, the full TNT degradation capacity of *E. coli* could only be understood if simultaneous activity of all pathways and the potential cooperation/trade-off between them is taken into account. To address this issue, we incorporated the complete reaction sets of NRP, DAP, and DNP into the metabolic model simultaneously to construct an in silico strain representing the major TNT degradation mechanisms in *E. coli*. FVA and RA were then used to explore the pattern and flexibility of flux distribution among multiple degradation pathways.

As seen from Table 2, under aerobic conditions *E. coli* replicates the GR and TNTUR when only DAP was present (see above), confirming preferred use of this pathway for aerobic degradation. However, at suboptimal condition, the flux structure gains flexibility to simultaneously activate DAP, DNP, and NRP as indicated by positive maximum fluxes through products of each pathway. This observation is supported by presence of NH_4_, NO_2_, and nitroreduction products in *E. coli* TNT-supplemented aerobic cultures [27,28]. Under optimal anaerobic conditions, again GR and TNTUR are similar to those when DNP was the only incorporated pathway, confirming the precedence of the nitrite-releasing route for anaerobic transformation. The excess NH_4_ produced by nitrite reductase is excreted as a by-product of TNT degradation. Under suboptimal anaerobic condition at the expanse of lower GR, flux can partition between DAP, DNP, and NRP, as represented by feasible excretion of NH_4_, NO_2_ and ADNT, respectively. Accordingly, while the optimal flux distribution is not flexible enough to allow simultaneous functioning of all pathways, under suboptimal conditions limited co-activity of multiple pathways is feasible.

**Figure 4:**
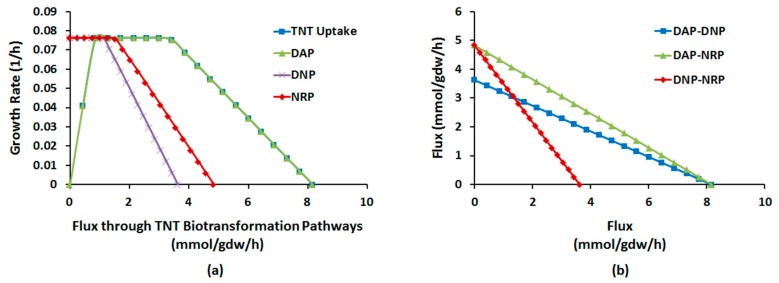
Robustness diagram: (**a**) aerobic growth rate (GR) as a function of fluxes through TNT biotransformation pathways; (**b**) pair-wise sensitivity of flux through each TNT degradation pathway to another under aerobic regime.

To gain a more dynamic insight into the trade-off between optimality and flux distribution flexibility, we performed robustness analysis between (1) GR and fluxes of DAP, DNP, and NRP; (2) fluxes of DAP, DNP, and NRP, pairwise, under both aerobic and anaerobic regimes. As Figure 4a illustrates, the plots of GR vs. TNTUR and DAP flux completely overlap, implying all uptaken TNT during aerobic growth goes preferentially through DAP. Beyond the maximum flux allowed by suboptimality (Table 2), GR begins to decline with all fluxes albeit at a different pace; the decrease in GR per unit of increase in flux is the highest for NRP, followed by DNP, and by far DAP. Therefore, aerobic biotransformation of TNT through NRP is most costly for the cell growth, whereas DAP imposes the lowest biomass cost.

As Figure 4b shows, the fluxes through DAP, DNP, and NRP have negative pairwise sensitivity with the order of DNP-NRP > DAP-DNP > DAP-NRP. Therefore, under aerobic regime DAP and NP can more easily cooperate to handle biotransformation of TNT whereas DNP and NRP represent the greatest trade-off.

**Figure 5:**
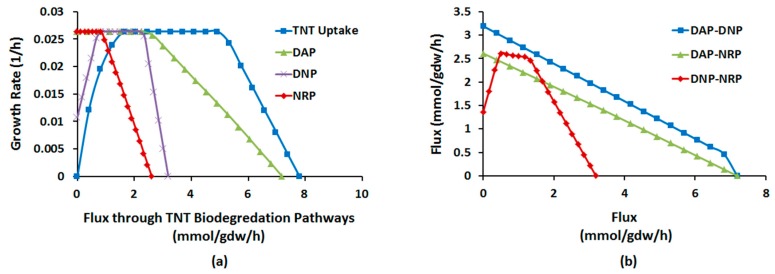
Robustness diagram: (**a**) anaerobic growth rate (GR) as a function of fluxes through TNT biotransformation pathways; (**b**) pair-wise sensitivity of flux through each TNT degradation pathway to another under anaerobic regime.

The relative behavior of optimal fluxes in anaerobic system shows remarkable differences. Figure 5a shows that contrary to aerobic conditions, growth at low TNTUR is coupled with DNP and insensitive to DAP activities. As DAP and DNP fluxes > ~2.2 mmol/gdw/h, GR begins to decrease with both, though with significantly higher pace with latter. The lower negative sensitivity of GR to DAP suggests that this pathway also allows more efficient transformation of TNT at high TNTUR under anaerobic condition.

According to Figure 5b, the sensitivity of DAP activity to DNP and NRP fluxes is similar and hence DAP can cooperate equally with both in carrying the uptaken TNT. The low activity of DNP is positively coupled to NRP flux. The coupling relaxes within DNP flux range of 0.5–1.3 mmol/gdw/h, after which the fluxes become competitive. Therefore while at low DNP activity, DNP-NRP pairs are co-active, such cooperation is difficult at high DNP flux.

The detailed description of competition or cooperation between TNT degradation pathways provided here can inform engineering of *E. coli* for efficient TNT biotransformation through the state-of-the-art systems and synthetic biology tools available for this strain [34,56].

### 2.2. Batch Culture Simulation

**Figure 6:**
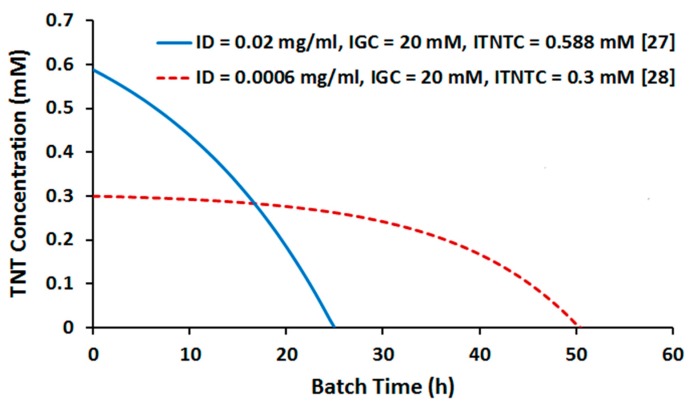
Batch time profile of TNT depletion as determined by dynamic flux balance analysis (DFBA) performed under experimental conditions.

Having explored the TNT transformation capabilities of *E. coli* at steady state, we extended our simulations to batch mode which is more practiced experimentally. Batch growth culture was simulated by dynamic flux balance analysis (DFBA) (see Materials and Methods). Sentuni et al. [27] and González-Pérez et al. [28] conducted batch incubation experiments to characterize *E. coli*’s ability to utilize TNT as the sole nitrogen source. To validate our in silico simulation, we conducted DFBA under the corresponding culture conditions. The study of Sentuni et al. [27] was carried out at inoculum density (ICD), initial glucose concentration (IGC), and initial TNT concentration (ITNTC) of 0.02 mg/mL (OD600 = 0.025), 20 mM, and 0.588 mM, respectively. The corresponding parameters in the experiment by González-Pérez et al. [28] were 0.0006 mg/mL, 20 mM, and 0.3 mM, respectively. Given the higher consistency of suboptimal in silico phenotypes with experimental data rather than optimal ones (see above), we maintained our assumption of 30% suboptimality during DFBA. The results are illustrated in Figure 6. As seen, TNT is depleted within 24 h of batch time in the first simulation and within 50 h in the second one. These results fairly compare with the observations of Sentuni et al. [27] and González-Pérez et al. [28] who reported disappearance of almost all TNT within 26 h and 48 h, respectively. This consistency of the DFBA results with experimental data validates the use of our simulation approach to explore optimal process parameters as will be discussed in the following sections.

### 2.3. Influence of Cultivation Condition on TNT Biotransformation Efficiency

Based on TNTURs reported in Table 2 (calculate at GUR = 250 mg/gdw/h), the yield of TNT biotransformation is estimated to be 1 and 3.49 (g/g) for optimal aerobic and anaerobic processes, respectively. Based on the yield comparison, hence, the anaerobic process turns out to be ~3.5 fold more efficient than the aerobic one. Nonetheless, the viability of a bioprocess relies not only on yield but on productivity as well [57]. While yield addresses the amount of products per unit of substrate consumed, productivity represents the amount of product per unit of time per unit of volume. The yield and productivity of batch/fed-batch bioprocesses often show trade-offs [57,58], making it difficult to achieve a high value for both. Given that productivity is highly influenced by GR [57,58] and anoxic GR is generally lower than oxic, the productivity of anaerobic vs. aerobic processes may not follow the yield pattern. In addition, apart from aeration regime there are also other parameters that can potentially influence yield and/or productivity, including initial TNT concentration (TNTIC), cell density (CD), and carbon source (CS). To gain a grasp of how these parameters would impact the efficiency of biodegradation, we conducted a series of constraint-based analyses as reported in the following sections.

#### 2.3.1. Effect of Oxygen Uptake 

**Figure 7:**
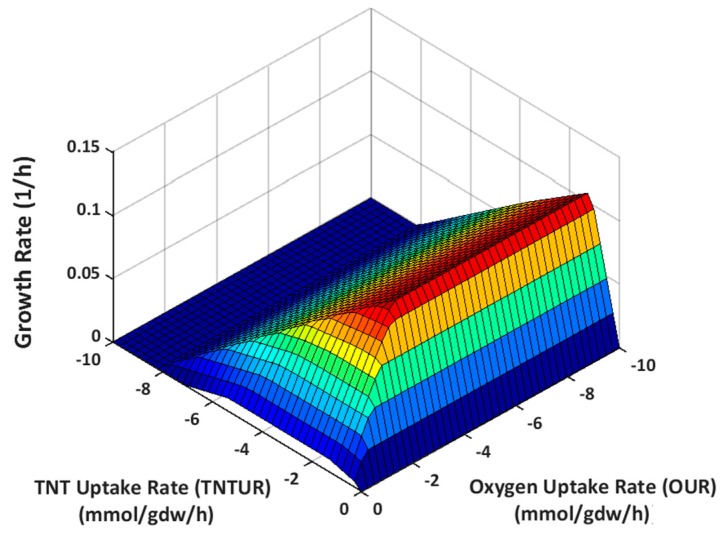
Phenotype phase plane: the growth rate is shown as function of TNTUR and OUR.

Oxygen uptake rate (OUR) is an important parameter in aerobic microbial processes [57,59,60,61,62]. The efficiency of aerobic bioremediation may be enhanced by adopting an appropriate aeration strategy [63,64,65]. CBA is frequently used to explore the relationship between OUR and microbial phenotypes of interest [60,63,66,67]. Herein, we used Phenotype Phase Plane (PhPP) Analysis to investigate how GR, OUR, and TNTUR are interrelated in *E. coli* when TNT is metabolized as the sole nitrogen source. To this end, we plotted the surface of optimal growth vs. OUR and TNTUR using the Phenotype Phase Plane function of the COBRA Toolbox [37]. According to Figure 7, at OUR > ~2.5 mmol/gdw/h the slope of line of global optimality is isocline to the oxygen uptake axis. However, below this level, the optimal growth phenotype is represented by a virtually triangular plane with positive slope along OUR and zero slope along TNTUR axes. This phenotypic phase change implies that at OURs below the maximum growth requirement TNTUR finds room to vary around the optimal value and the variation span becomes wider with OUR decrease. According to the PhPP diagram, a decrease of OUR down to 60% of the maximum growth requirements can raise TNTUR up to 1.5-fold while retaining 75% of the optimal growth. In another scenario, constraining OUR to 40% of optimal value increases the maximum feasible TNTUR by 2-fold at 50% of optimal growth.

**Figure 8:**
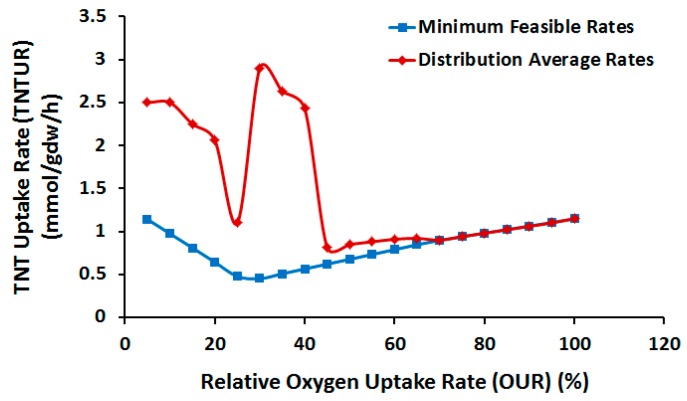
TNT Uptake Rate (TNTUR) as a function of oxygen uptake rate (OUR) relative to the optimal value (%).

Despite this potential, improvement of TNTUR by suboptimal aeration is not guaranteed because at similar GR, TNTUR has the room to approach the other edge of the phenotype plane, taking values lower than the optimal growth conditions. To alleviate the uncertainty concerning actual position of flux we randomly sampled the flux space at various OUR levels. Figure 8 shows the average and minimum TNTUR as a function of relative OUR (relative to the optimal value (%)). Interestingly, average TNTUR shows a highly non-linear relationship with OUR. The highest TNTUR level is seen at OUR = 30% of the optimal value, followed by 35% and 40%. Below this range a large decrease is observed which is considerably compensated when OUR inclines towards a microaerobic regime. Above the aforementioned range TNTUR falls to the lower limit of the feasible interval. The nonlinear response of TNTUR to linear variation of OUR reflects the complex nature of TNT metabolism and the need for systems approach to identify optimal biodegradation process parameters. Based on our results the ideal OUR range for TNT biotransformation is 30–40% of optimal value, which at the same time preserves 43–54% of maximum GR.

**Figure 9:**
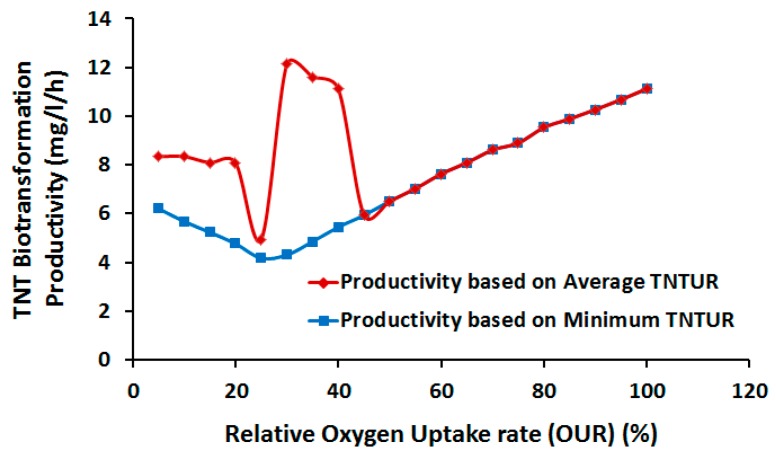
TNT biotransformation productivity as a function of oxygen uptake rate (OUR) relative to the optimal value (%).

As mentioned earlier, the low aeration regime, while possibly enhancing biotransformation rate, may negatively impact the productivity due to reduced GR. To examine whether the low-OUR-induced improvement in TNTUR would be followed by productivity, using DFBA we computed the productivity vs. relative OUR at the average (and minimal) TNTUR values. As comparison of Figure 8 and Figure 9 indicates, the variation of average productivity with OUR is consistent with that of TNTUR, confirming the suitability of relative OUR of 30–40% for biotransformation process. Although according to Figure 9 a full aeration regime allows for comparable productivity, the abovementioned OUR range is also supportive of a comparatively higher yield.

#### 2.3.2. Effect of TNT Concentration

**Figure 10:**
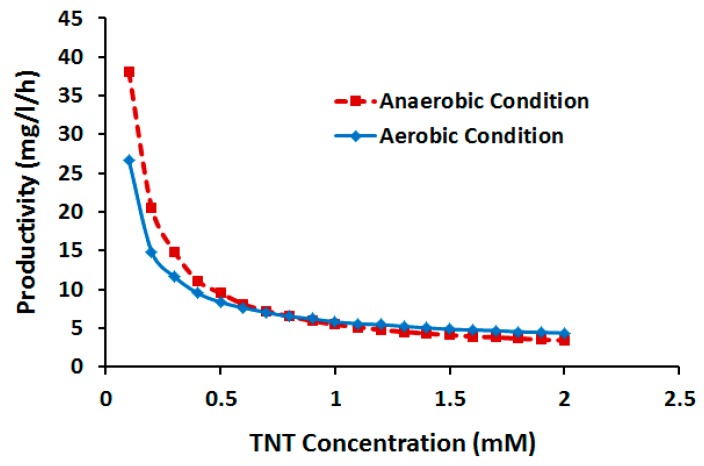
Productivity of TNT biotransformation vs. concentration.

The efficiency of microbial remediation of NACs is constrained by two major factors: (1) low rate of degradation hampering time-efficient clean-up of contaminants (particularly at high loading rates); and (2) the relatively low tolerance of microbes to chemical toxicity [3]. Studies on the *E. coli* TNT tolerance have reported various multiple thresholds ranging from 66 to 200 mg/L [31,32]. However, there is meager information on how variation of initial TNT concentration within the nontoxic range may affect degradation efficiency. To gain insight into this, we conducted DFBA at various initial TNT concentrations up to the maximum toxic threshold [31], under both aerobic and anaerobic regimes. As shown in Figure 10, lower TNT concentrations are correlated with higher productivity. Also at lower concentrations productivity is higher in anaerobic vs. aerobic processes. On the other hands, as concentration exceeds 0.8 mg/mL (typical values in batch cultivation [27,31]), productivity tend to lose negative sensitivity to both concentration and aeration mode, approaching a constant level. Low sensitivity of productivity to concentration is advantageous because it permits increased biotransformation yield (by operating at high TNT concentration) with limited negative effect on productivity. The stability of productivity against concentration is also indicative of the adaptability of *E. coli* to variation in chemical loading rate. Both of these advantages however are constrained to the maximum toxic concentration threshold, highlighting the importance of improved TNT tolerance for viable biodegradation processes.

#### 2.3.3. Effect of Cell Density

**Figure 11:**
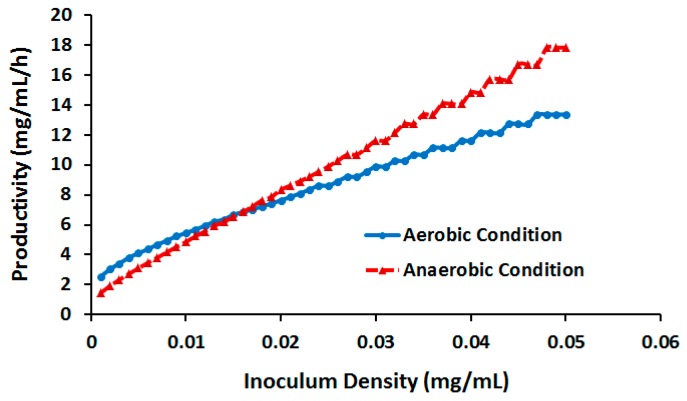
Productivity of TNT biotransformation vs. inoculum density.

Although *E. coli* generally grows slower under anoxic compared with oxic regimes [68], we observed a higher productivity of anaerobic culture at low concentrations and similar productivity of both processing mods at high TNT. This can be partly justified by the comparatively high TNTUR under anoxic regimes due to the activity of nitrite reductase (see above), compensating the effect of reduced GR. However, one must note that this result was obtained by assuming identical inoculum density (ID) at both cultivation conditions, whereas anaerobic bacterial inocula generally present lower density [68,69]. While the effect of ID on TNT degradation has been examined in some of bacteria such as *Pseudomonas* sp. strain TM15 [52], no such evaluation has been published for *E. coli*. Therefore we examined in silico the impact of ID on biodegradation productivity. In doing so, DFBA was performed at various ID levels around the experimentally examined value of 0.02 and fixed TNT concentration of 0.588 mM. As illustrated in Figure 11, the productivity of both processes shows a virtually linear correlation with ID, with the anaerobic process productivity presenting a larger slope. In addition, while at low IDs productivity is lower in an anaerobic than an aerobic process, as ID exceeds 0.016 the productivity of the former prevails. Overall, our analysis shows that productivity of anaerobic processes is more sensitive to ID so that at sufficiently high IDs, the anaerobic process has the potential to outcompete an aerobic process.

Due to the advantages of aerobic cultures such as rapid growth rate and diverse metabolism, high CD aerobic processes have traditionally attracted more interest compared with anaerobic processes. Notwithstanding, aerobic cultivation has its own drawbacks when applied to TNT bioremediation, including: (1) the formation of more toxic and recalcitrant compounds than TNT (such as azoxytetranitrotoluenes) in the presence of oxygen; (2) limited biodegradation rate because of (1), and (3) low availability of TNT metabolites to biodegradation due to their binding to the organic soil materials [1,3,9,13].

To avoid these limitations, in recent years much attention has been been focused on anaerobic biodegradation [1,9,13,20], as witnessed by the fact that anoxic treatment represents the major process step in current ex situ TNT bioremediation technologies [3,13]. According to our results, high CD anaerobic process would combine the advantages of high productivity and minimal production of recalcitrant intermediates, thus deserving further research and development efforts.

#### 2.3.4. Effect of Alternative Carbon Sources

**Figure 12:**
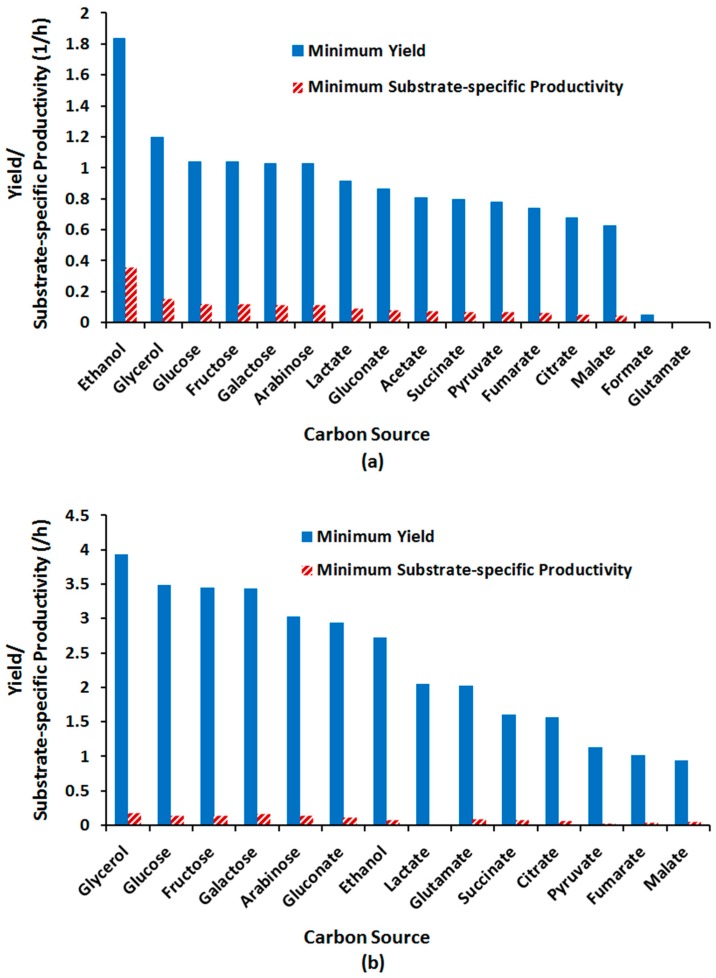
Assimilatory TNT biotransformation yield for various carbon sources as (co)substrate under (**a**) aerobic and (**b**) anaerobic growth regimes.

Co-metabolic biotransformation of nitroaromatics requires the presence of primary substrate(s) serving as sources of carbon and electrons [3,9,13]. Given that different carbon sources (CSs) are catabolized through bioenergetically distinct metabolic pathways, the choice of substrate may affect the capacity of metabolism for the desired phenotype. The influence of feeding regime on the efficiency of microbial TNT transformation has been demonstrated [22,23,24,70]. Several attempts have been made to improve the rate of TNT biodegradation by identifying appropriate carbon and energy sources [23,24]. Most of these investigations, however, are limited to a few number of substrates and supplementary nutrients. CBA of GSMMs provides an excellent tool to identify and rank the most efficient carbon/energy sources and thereby optimal feeding scenarios for the bioprocess of interest. Aggarwal et al. used CBA to compare the relative suitability of various carbon, nitrogen, and sulphur sources for desulphurization by *Rhodococcus erythropolis* [42]. Nambou et al. [71] showed that CBA-assisted medium design can lead to higher lycopene titres in an engineered *Yarrowia lipolytica* strain compared with CBA-independent culture optimization. In the present study, we applied CBA to identify CSs inducing the highest yield of TNT bioconversion in *E. coli*. For this purpose, compounds including glucose, fructose, galactose, arabinose, glycerol, ethanol, pyruvate, acetate, citrate, fumarate, succinate, malate, lactate, gluconate, and glutamate were separately set as the limiting substrate of *E. coli* TNT-supplemented growth culture, followed by FBA and FVA to calculate rate and thereby yield of biotransformation. The effectiveness of alternative substrates on yield of assimilatory TNT transformation is compared in Figure 12. As seen, under aerobic growth condition, ethanol allows the highest biotransformation yield, followed by glycerol and glucose/fructose/galactose/arabinose (Figure 12a). These substrates also support the highest substrate-specific productivity (yield × growth rate) in that respective order. Under anaerobic conditions, acetate and formate could not support growth on TNT as the sole nitrogen source. Among the growth-supporting carbon sources, glycerol turned out to be the most effective substrate for TNT degradation, followed by glucose/fructose/galactose, and arabinose/gluconate (Figure 12b). In a different order, glycerol, followed by galactose, and glucose/fructose/arabinose induced the highest substrate-specific productivity.

According to CBA predictions, therefore, ethanol and glycerol are identified as the most effective substrates for aerobic and anaerobic TNT degradation processes, respectively. The superiority of these substrates lies in generation of a comparatively larger pool of reducing equivalent per carbon flux, to be used in TNT degradation pathways. Oxidation of both compounds to central metabolites requires two oxidized equivalents: two NAD^+^s for ethanol and a NAD^+^ and a NADP^+^ for glycerol. Under an aerobic growth regime where NAD^+^ is abundant due to the activity of oxidative phosphorylation, oxidation of ethanol will be more efficient than that of glycerol. Conversely, during anoxic growth where NAD^+^ is more limited than NADP^+^, glycerol oxidation which needs lower amounts of NAD^+^ is preferred.

### 2.4. Metabolic Engineering Targets for Improved Biotransformation of TNT

Metabolic engineering (ME) finds an increasing use in bioremediation process development [72,73,74,75]. In the nitroaromatics bioremediation area, several effective gene manipulations have been reported [3]. For example, heterologous expression of pentachlorophenol from *Sphingomonas* strain UG30 (pcpB) to *E. coli* enabled the strain to degrade 4-nitrocatechol and *p*-nitrophenol concomitantly with nitrite release [76]. In another effort, heterologous expression in *E. coli* of recombinant plasmids from *Enterobacter cloacae* and *P. pseudoalcaligenes* JS45 carrying nitrobenzene reductase (nfs1) and nitrobenzene nitroreductase (nbzA), respectively, resulted in rapid conversion of nitrobenzene to aminophenol (64%) [77].

**Figure 13:**
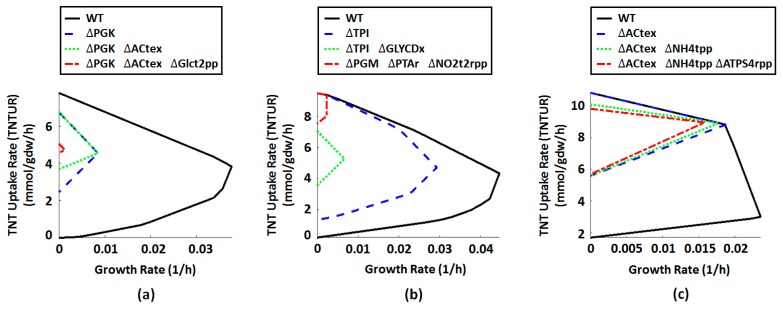
Growth envelope for various TNT uptake strain designs during growth on (**a**) glucose; (**b**) glycerol; and (**c**) ethanol.

Rational design of microbial agents for bioremediation is boosted by the advent of GSMMs, enabling comprehensive analysis of the strains’ capabilities in degradation of toxic compounds [39,40,41,42,78]. We used this advantage to identify ME targets potentially improving the specific rate of TNT biodegradation. To this end, we separately conducted single, double, and triple reaction deletion experiments in glucose-, glycerol-, and ethanol-limited media and evaluated the TNT transformation capability of the obtained mutants. Single-reaction-knockout (KO) experiments were conducted using the simpleOptKnock function implemented in the COBRA Toolbox checking all single reaction deletions for growth-coupled metabolite production/consumption. Double- and triple- KO design were conducted by the RobustKnock algorithm, developed to maximize the minimum flux of a target bioconversion while maximizing the growth rate. Our criterion for considering a particular KO scenario as an effective one is that the relevant in silico mutant shows higher minimum feasible TNTUR compared with the wild-type (WT) strain. This criterion implies that the KO strategy should force the cell to uptake TNT more rapidly in order to realize the maximum growth. Under aerobic growth condition, no effective strain design scenario was identified based on single- to triple- KO strategy. However, under anaerobic regime, in silico ME introduced several promising designs. Table 3 shows the list of reactions in all effective KO experiments and Figure 13 and Table S1 present strain designs on glucose, glycerol and ethanol media.

The most efficient strain designs were obtained for growth on ethanol. As seen from Figure 13 and Table S1, ∆ACtex-∆ACtex ∆NH4tpp and ∆ACtex −∆NH4tpp −∆ATPS4rpp mutants grown on ethanol are forced to uptake TNT by nearly 3-fold as high as in the WT strain while retaining 83%, 78%, and 70% of the optimal WT growth rate, respectively. In glycerol-limited medium, while the single KO design shows a 9% improvement in TNTUR, the double KO design represents 21% higher TNTUR relative to the WT strain at 15% of its GR. At the same time, the triple KO mutant ∆PGM-∆PTAr-∆NO_2_t2rpp shows high TNTUR (comparable with ethanol-medium-based designs) at the expanse of very low GR (5% of WT). Strains designed in glucose-limited medium show the least efficiency among all. However, the single KO (∆PGK) and double KO (∆PGK-∆ACtex) designs present comparable phonotypes with ∆TPI-∆GLYCDx mutant in glycerol-limited medium. Congruent with carbon source effectiveness study (Section 2.3.3), the results of in silico strain design suggests ethanol as the best substrate for enhanced TNT biotransformation through ME. These findings may guide future experimental efforts in developing efficient TNT degradation bioprocesses and encourage extension of such studies to more complex biosystems including co-culture systems [79], and natural/synthetic microbial communities [80].

## 4. Materials and Methods

### 4.1. Model Preparation

The genome-scale metabolic model (GSMM) of an organism is a mathematical representation of its metabolism in silico. Ideally, the entire known metabolic reaction network is modelled as a stoichiometric matrix S with rows addressing the metabolites and columns addressing the reactions. Each element of Sij in the stoichiometric matrix is the coefficient of the ith metabolite in the jth reaction. Each reaction is linked to its catalysing enzyme which in turn is associated with its encoding gene(s). The relationship between genes, proteins, and reactions are established based on Boolean logical regulatory rules. The structure of metabolic network hence captures essential molecular biology of the relevant organism at metabolic level.

The genome-scale metabolic network is further developed to a constraint-based model (CBM) by restricting the flux of each reaction to physico-biologically relevant constraints. Typical constraints imposed on the fluxes include unidirectionality of some reactions, quasi-steady-state metabolic operation, and optimal flux distribution. The CBM can then be explored by constrain-based analysis (CBA) to characterize metabolic phenotypes under defined environmental conditions.

In the present study we used the most comprehensive high quality metabolic model of *E. coli*, iJO1366, to analyse the phenotypes relevant to TNT metabolism in this bacterium. In doing so, firstly we extended iJO1366 to account for a curated version of the known or evidently hypothesized pathways of TNT biotransformation in *E. coli*. The pathway information was extracted from literature [1,2,3,9,13,27,28,29,30,44,45]. Each reaction was examined for being elementary and charge balanced using modelGeneration functions of COBRA Toolbox [37] and was manually balanced whenever necessary. Figure 1 illustrates the pathways added to the model and the complete list of all incorporated reactions is provided in Supplementary Materials File 1.

### 4.2. Constraint-Based Analysis

We adopted a constraint-based approach to analyse the TNT biotransformation capacity of *E. coli*. The standard protocol for constraint-based analysis (CBA) of metabolic networks is documented [37]. Given the objective of our study we used among the diverse set of CBA techniques [37], the Flux Balance Analysis (FVA), Flux Variability Analysis (FVA), Flux Distribution Sampling, Robustness Analysis (RA), and Phenotype Phase Plane (PhPP) Analysis to explore the *E. coli* extended GSMM.

#### 4.2.1. Flux Balance Analysis

Because each metabolite usually participates in more than one bioconversion, the metabolic network is an underdetermined system and thus there will be an infinitive number of solutions to satisfy the stoichiometric constraints. FBA is a linear-programing-based approach to find one steady-state flux distribution solution within the closed flux space allowing the global optimal value of a specified metabolic objective function (OF) to be reached [37,81]. Based on the evidence that under the evolutionary pressure the metabolism has been enforced to support the maximum growth rate [82], maximization of biomass production is typically considered at the OF of the metabolic models. Thereby, the FBA problem in our work was formulated as the following:

$$\max c^{T}v,\;\mathrm{Subject}\;\mathrm{to}\;S_{ij}\cdot v_{j}=0,\;v_{j}^{min}\leq v_{j}\leq v_{j}^{max}$$

#### 4.2.2. Dynamic Flux Balance Analysis

While FBA estimates the optimal growth based on quasi-steady-state assumption, the microbial batch growth is a dynamic process in which biomass concentration increases exponentially along gradual exhaustion of the limited substrate. Dynamic flux balance analysis (DFBA) is an iterative FBA-based method for simulation of the (fed-)batch bioprocess [68]. According to DFBA, substrate and biomass concentrations can vary over a series of discrete time steps at each of which the intracellular metabolism would operate quasi-steadily [68]. The assumption of quasi-steady-state metabolic operation in spite of varying extracellular concentrations is based on the experimentally valid premise that enzymatic bioconversions are fast enough to adapt virtually immediately to bioprocess dynamics [83]. For DFBA, at each time step we applied FBA to estimate growth, glucose, and TNT uptake rates. The obtained rates were then used for calculating cell and TNT concentrations (based on standard differential equations describing batch microbial growth and substrate disappearance) and thereby maximum glucose and TNT uptake rates for the next time step [68]. The procedure was repeated at each new time step until the TNT was exhausted. The DFBA was conducted under batch incubation experimental conditions [27,28].

#### 4.2.3. Flux Variability Analysis

While the LP nature of FBA guarantees identification of the globally optimal OF value (here biomass production), this unique value could be realized through infinitive number of flux distributions. In another word, flux through many reactions can vary within a certain range without affecting OF. FVA is a FBA-based technique to identify the span within which each reaction flux can vary while still supporting the FBA-determined OF value [37,84].

$$\max/\min v_{j},\;\mathrm{Subject}\;\mathrm{to}\;S_{ij}\cdot v_{j}=0,\;c^{T}v=Z_{biomass}\;v_{j}^{min}\leq v_{j}\leq v_{j}^{max}$$

Comparison of in silico and experimental data in our and some previous studies shows that the optimal metabolic operation assumed by FBA is usually not realized during wet-lab experiments [27,28]. The suboptimal activity of metabolic reactions can computed by performing FVA under the situation that the biomass production is restricted to values below the maximum [84]. In the present study a constraint of 70% of the optimal biomass production rate was assumed for all suboptimal flux calculations.

#### 4.2.4. Sampling of Solution Space

Although all reaction flux values within FVA-determined interval will yield an identical OF value, the probability distribution of flux within the feasible range is not uniform. Radom sampling of the flux space can elucidate the structure of flux distribution which in turn can be analyzed to obtain the most probable flux values within the allowable space [46]. Whenever informative, we sampled the flux space at 5300 random points (more than 2 × number of extended model reactions) using Monte Carlo algorithm [37,46]. The flux distribution of the reactions of interest was then inspected for skewness, and the mean, standard deviation (SD), and median of each flux was calculated.

#### 4.2.5. Robustness Analysis

Robustness analysis (RA) is a CBA technique to evaluate the amount with which a metabolic OF will change along with a unit of change in a particular reaction flux (control flux) [50]. The RA is calculated by stepwise constraining of the control flux within its feasible range and performing FBA at each step. The obtained RA diagram thereof will inform about the sensitivity of the objective flux to variation of control flux [85].

#### 4.2.6. Phenotype Phase Plane Analysis

The FBA-determined optimal flux distribution is a function of environmental parameters imposed on the metabolic network. Phenotype Phase Plane (PhPP) analysis is used to characterize how an optimal phenotype of interest will response to variation of environmental conditions [86]. We used PhPP analysis to delineate the response of GR to change in oxygen uptake rate (OUR) and TNT uptake rate (TNTUR). Both of these parameters could be externally set by controlling availability of the respective substances during cultivation.

### 4.3. In Silico Strain Design

Genome-scale constraint-based metabolic models are extensively used to identify relevant metabolic engineering strategies aimed at improved yield of a target bioconversion [33,87]. We used the extended GSMM of *E. coli* to predict a series of strain design scenarios enabling higher yield of TNT biotransformation. More specifically, our aim was to identify single- to triple-reaction knockouts (KOs) potentially improving the minimum feasible TNTUR relative to the wild-type (WT) strain. Several CBA-based methods have been developed for in silico strain design [88]. OptKnock was the first bi-level strain optimization algorithm to maximally improve the flux of a target bioconversion while maximally maintaining cell viability [88,89]. Due to redundancy of flux distribution over the metabolic network, OptKnock cannot guarantee the improved yield of the target product in vivo because flux may be directed towards competing pathways with no cost for growth [90]. A tri-level algorithm RobustKnock was subsequently proposed to eliminate this limitation by taking into account presence of all competing pathways, enabling maximization of the minimum yield of the target bioconversion [90]. Because our aim was to improve the minimum TNTUR, for single-KO expriment the lower and upper bounds of each reaction flux was sequentially set to zero and the minimum TNTUR required by optimal growth was calculated (using simpleOptKnock function of the COBRA Toolbox [37]), and for the double- and triple-KO experiments the RobustKnock algorithm was employed. The results of in silico ME was illustrated by plotting growth envelope for each designed strain.

## 5. Conclusions

The present study extended the application of constraint-based analysis (CBA) to the microbial remediation of nitroaromatic compounds (NACs). Our results replicated several TNT biotransformation phenotypes observed in *E. coli*, including control of nitroreduction pathway by NADPH availability, extreme limitation of complete TNT nitroreduction, simultaneous release of nitrite, ammonium, and nitro reduction products during TNT degradation, and the time profile of TNT elimination in batch cultures. Our analyses also provided potentially useful insights into the impact of process parameters on TNT bioremediation efficiency, including oxygen uptake rate (OUR) and aeration regime, TNT concentration, inoculum density, and carbon source. High cell density ethanol-limited anaerobic cultivation showed the highest efficiency among other processes, giving it merit for more in-depth research. We also designed a series of *E. coli* stains with improved TNT biotransformation phenotype in silico. The obtained results may guide future efforts in development of high performance microbial TNT clean-up process and highlights CBA as an informative approach to accelerating bioremediation research.

## Figures and Tables

**Table 1:** Variability, mean, and median of TNT uptake rate (TNTUR) and fluxes through reduced metabolites.

Compound	Aerobic Conditions			Anaerobic Conditions		
	Variability (mmol/gdw/h)	Mean (SD) (mmol/gdw/h)	Median (mmol/gdw/h)	Variability (mmol/gdw/h)	Mean (SD) (mmol/gdw/h)	Median (mmol/gdw/h)
TNT	[0, 1.5]	0.01 (0.048)	0.004	[0, 0.9]	0.45 (0.41)	0.45
ADNT	[0, 1.5]	0.008 (0.036)	0.002	[0, 0.9]	0.41 (0.039)	0.42
DANT	[0, 75]	0.003 (0.017)	0	[0, 0.45]	0.033 (0.043)	0.022
TAT	[0, 0.5]	0.002 (0.012)	0	[0, 0.3]	0.004 (0.011)	0.001

**Table 2:** Variability of TNT uptake rate (TNTUR) and flux through TNT degradation products under different cultivation conditions.

Cultivation Condition	Growth Rate	TNT Uptake Rate	NH_4_ Production Rate	NO_2_ Production Rate	ADNT Production Rate
Aerobic	0.11	[1.15, 1.15]	[0, 0]	[0, 0]	[0, 0]
Suboptimal Aerobic	0.076	[0.8, 3.36]	[0, 2.56]	[0, 1.12]	[0, 1.5]
Anaerobic	0.038	[3.84, 3.84]	[1.52, 1.52]	[0, 0]	[0, 0]
Suboptimal Anaerobic	0.026	[1.46, 5.11]	[0.18, 3.44]	[0, 0.64]	[0, 0.85]

**Table 3:** Relevant reaction in TNT biotransformation strain design.

ID	Name	Formula	Gene Association
PGK	Phosphoglycerate kinase	3pg[c] + atp[c] <=> 13dpg[c] + adp[c]	b2926
Actex	Acetate transport via diffusion (extracellular to periplasm)	ac[e] <=> ac[p]	(b0241 or b0929 or b1377 or b2215)
GLCt2pp	D-glucose transport in via proton symport (periplasm)	glc-D[p] + h[p] -> glc-D[c] + h[c]	b2943
NH4tpp	Ammonia reversible transport (periplasm)	nh4[p] <=> nh4[c]	(b0451 or s0001)
TPI	Triose-phosphate isomerase	dhap[c] <=> g3p[c]	b3919
GLYCDx	Glycerol dehydrogenase	glyc[c] + nad[c] -> dha[c] + h[c] + nadh[c]	b3945
NO2t2rpp	Nitrite transport in via proton symport, reversible (periplasm)	h[p] + no2[p] <=> h[c] + no2[c]	(b3367 or b1223)
PGM	Phosphoglycerate mutase	2pg[c] <=> 3pg[c]	(b3612 or b4395 or b0755)
PTAr	Phosphotransacetylase	accoa[c] + pi[c] <=> actp[c] + coa[c]	(b2297 or b2458)
PPS	Phosphoenolpyruvate synthase	atp[c] + h2o[c] + pyr[c] -> amp[c] + 2 h[c] + pep[c] + pi[c]	b1702
ATPS4rpp	ATP synthase (four protons for one ATP) (periplasm)	adp[c] + pi[c] + 4 h[p] <=> atp[c] + 3 h[c] + h2o[c]	(((b3736 and b3737 and b3738) and (b3731 and b3732 and b3733 and b3734 and b3735) and b3739) or ((b3736 and b3737 and b3738) and (b3731 and b3732 and b3733 and b3734 and b3735)))

## Supplementary Materials

The following are available online. Supplementary File 1: the complete list of TNT biotransformation reactions included in this study. Supplementary File 2: detail characteristics of the in silico designed strains.

## Abbreviations

CBA	constraint-based analysis
GSMM	genome-scale metabolic model
CBM	constraint-based model
FBA	flux balance analysis
DFBA	dynamic flux balance analysis
FVA	flux variability analysis
RA	robustness analysis
PhPP	phenotype phase plane
NACs	nitroaromatic compounds
TNT	2,4,6,-trinitrotoluene
NDTs	nitrosodinitrotoluenes
ADNTs	aminodinitrotoluens
HADNTs	hydroxylaminodinitrotoluenes
DANTs	diaminonitrotoluenes
NODNT	2-nitroso-4,6-dinitrotoluene
HADNT	2-hydroxylamino-4,6-dinitrotoluene
ADNT	2-amino-4,6-dinitrotoluene
ANONT	2-amino-4-nitroso-6-nitrotoluene
HANT	2-amino-4-hydroxylamino-6-nitrotoluene
DANT	2,4-diamino-6-nitrotoluene
NOAT	6-nitroso-2,4-diaminotoluene
HTAT	6-hydroxylamino-2,4-diaminotoluene
TAT	2,4,6-triaminotoluene
DHDNT	2,5-dihydroxy-4,6-dinitrotoluene
AHDNT	2-amino-5hydroxyl-4,6-dinitrotoluene
3h-tnt	TNT-monohydride complex [(3-H-)-TNT]
3,5,2h-tnt-h	TNT-dihydride complex [(3,5-2H-)-TNT.H+]
N,N(3,5-DNT)amine	*N*,*N*-bis(3,5-dinitrotolyl) amine
NRP	nitroreduction pathway
DAP	deamination pathway
DNP	denitration pathway
GR	growth rate
GUR	glucose uptake rate
IGC	initial glucose concentration
OUR	oxygen uptake rate
TNTUR	TNT uptake rate
ITNTC	initial TNT concentration
CD	cell density
ID	inoculum density
CS	carbon source
OF	objective function
KO	knockout
WT	wild-type
ME	metabolic engineering
