# Supplementary material for: Systems Biology Approach to Bioremediation of Nitroaromatics: Constraint-Based Analysis of 2,4,6-Trinitrotoluene Biotransformation by Escherichia coli
Source: Molecules. 2017 Aug 14;22(8):1242. doi: 10.3390/molecules22081242 (PMC6152126; doi:10.3390/molecules22081242)
Supplement: Supplementary file 1 [file molecules-22-01242-s001.zip › Supplementary File 2.docx]

**Table S1** Comparison of wild-type and *in silico* designed mutant strains in growth rate and TNT uptake rate (TNTUR) during growth on (**a**) glucose, (**b**) glycerol, and (**c**) ethanol.

| **Carbon source** | **Design strategy** | **Wild-type/Mutant Strain** | **Growth rate (1/hr)** | **Min TNTUR**  **(mmol/gdw/hr)** | **Max TNTUR**  **(mmol/hdw/hr)** |
| --- | --- | --- | --- | --- | --- |
| **Glucose** |  | Wild-type | 0.038 | 3.84 | 3.84 |
|  | Single KO | ∆PGK | 0.009 | 4.56 | 4.56 |
|  | Double KO | ∆PGK, ∆Actex | 0.0086 | 4.56 | 4.56 |
|  | Triple KO | ∆PGK, ∆Actex, ∆GLCt2pp | 0.0013 | 4.73 | 4.73 |
| **Glycerol** |  | Wild-type | 0.045 | 4.33 | 4.33 |
|  | Single KO | ∆TPI | 0.029 | 4.71 | 4.71 |
|  | Double KO | ∆TPI, ∆GLYCDx | 0.0067 | 5.26 | 5.26 |
|  | Triple KO | ∆PGM, ∆PTAr, ∆NO2t2rpp | 0.0024 | 8.04 | 9.4 |
| **Ethanol** |  | Wild-type | 0.023 | 3.00 | 3.00 |
|  | Single KO | ∆ACtex | 0.019 | 8.84 | 8.84 |
|  | Double KO | ∆ACtex, ∆NH4tpp | 0.0018 | 8.95 | 8.95 |
|  | Triple KO | ∆ACtex, ∆NH4tpp, ∆ATPS4rpp | 0.0016 | 8.99 | 8.99 |
